# Vascular Endothelial Growth Factor (VEGF) Gene Promoter Polymorphisms and Disease Progression in North Indian Cohort with Autosomal Dominant Polycystic Kidney Disease

**DOI:** 10.22088/acadpub.BUMS.6.3.164

**Published:** 2017-09-26

**Authors:** Shewata Pandita, Deepshikha Maurya, Vijaya Ramachandran, Jyotsna Verma, Sudha Kohli, Renu Saxena, Ishwar Chander Verma

**Affiliations:** 1 *Institute of Medical Genetics and Genomics, Sir Ganga Ram Hospital, Rajinder Nagar, New Delhi, India.*; 2 *Guru Gobind Singh Indraprastha University, Dwarka, New Delhi, India.*; 3 *Current address: South West Thames Regional Genetics Laboratory, St. George’s University Hospitals NHS Foundation Trust, London SW17 0QT, United Kingdom.*

**Keywords:** *ADPKD*, *VEGF* gene, chronic kidney disease, haplotype, North Indian

## Abstract

Autosomal dominant polycystic kidney disease (ADPKD) is characterized by a significant phenotypic variability in progression of the disease. Vascular endothelial growth factor (*VEGF*) has been reported to play a major role in renal pathophysiology. The aim of the present case-control study was to evaluate the association of two promoter polymorphisms (-2578C>A and-1154G>A) of *VEGF* gene and ADPKD. Genotyping was carried out in 123 ADPKD patients and 100 healthy controls, using a polymerase chain reaction-restriction fragment length polymorphism technique (PCR-RFLP). The genotype, allele and haplotype frequencies of these two polymorphisms in ADPKD patients were compared with those in controls, as well as in patients with early and advanced chronic kidney disease (CKD) stages, using Chi-square (χ2) test. The distribution frequency of CC, CA and AA genotypes of -2578C>A polymorphism differed significantly between patients and controls (0.31, 0.63 and 0.06 vs 0.37, 0.44 and 0.19, respectively (P=0.003)), but no significantly different genotype distribution was observed for the-1154G>A polymorphism. The A allele of -2578C>A and G allele of -1154G>A, were significantly more present in the controls as compared to the patients, and may provide protection for CKD under recessive (OR, 3.73; 95% CI, 1.45-9.62; P=0.0042), and dominant (OR, 0.55; 95%CI, 0.31-0.98; P=0.041) models. The [A;G] haplotype was more frequently present in controls (18%) than in cases (8%), (OR 0.398; 95% CI 0.22-0.71; P=0.002). These results suggest that the two promoter polymorphisms of *VEGF* may modify the disease risk in ADPKD patients from North India.

Autosomal dominant polycystic kidney disease (ADPKD) is the most common hereditary renal disease characterized by the development of multiple fluid-filled cysts derived from renal tubule epithelial cells. These cysts keep increasing in size and number and gradually cause renal failure in about 50% of the ADPKD patients by the age of 60 years (1). Data from Chronic Kidney Disease (CKD) Registry of India reveals that ADPKD accounts for 2.5% of CKD in India (2). The severity of the disease, the age of onset of end stage renal disease (ESRD) and the extra-renal manifestations vary widely among the affected individuals. In 85% of the patients, a mutation is observed in *PKD1* gene, while the rest have mutations in *PKD2* gene (3). Generally, the mean age of ESRD in *PKD1* linked patients is 54 years and in *PKD2* linked patients is 70 years (4). The variability in the clinical course of the disease among individuals could be due to the gene involved, type of mutations in PKD genes or the protein domain that is involved. However, inter-familial variability cannot be fully explained on this basis, and may be due to other genetic and environmental modifiers (5).

The pathophysiology of ADPKD includes abnormal proliferation of renal epithelial cells, an increase in the extracellular matrix, and vascular alterations due to the expansion of cysts. This is a highly active process and requires oxygen and nutrients for processes like expansion and fluid secretion. Bello-Reuss et al. examined the kidneys from 14 patients with ADPKD and identified extensive capillary network in the cyst walls along with vascular malformations (6). They also reported that vascular endothelial growth factor-165 (VEGF_165_) was expressed in cysts cells and VEGF receptor 2 (VEGFR-2) proteins in capillaries surrounding the cysts and in glomeruli. VEGF, also known as vascular permeability factor (VPF) is a potent angiogenic agent that stimulates endothelial cell proliferation and differentiation, increases vascular permeability, and mediates endothelium dependent vasodilatation. The role of VEGF protein in renal pathophysiology is well established in diabetic nephropathy, glomerulonephritis, hemolytic uremic syndrome, and various other renal diseases (7). In normal adult kidneys, VEGF and VEGFR-2 are expressed in the visceral epithelial cells and in the endothelia of glomeruli, respectively. Weak expressions of VEGF in collecting ducts and VEGFR-2 in the surrounding capillaries have also been observed (8). These findings suggest that VEGF is involved in the development of peri-cystic circulation and hence, provides support for growth of the cysts.

The *VEGF* gene is located on chromosome 6 (6p21.1). It is highly polymorphic, especially in the promoter, 5’-untranslated and 3’-untranslated regions (9). Some of these polymorphisms (-2578C>A, -1154G>A, -634G/C and +936C/T) have been related to varying VEGF protein expression and serum VEGFA levels (10,11), in various diseases, such as renal cell carcinomas (12), recurrent pregnancy loss (13), breast cancer (9) and lung cancer (14). The polymorphisms, -2578C>A (rs699947) and -1154G>A (rs1570360) have been documented to influence the progression of disease in ADPKD patients (15). The two VEGF polymorphisms had a minor allele frequency (MAF)≥ 0.05, ascertained from South Asian (SAS) population in 1000 Genomes Phase 3 data (http:// phase3browser.1000genomes.org/index.html). In the present study we analyzed the distribution of -2578 >A and -1154G >A promoter polymorphisms of *VEGF* gene in patients of ADPKD and controls, and also examined their association with the progression to chronic kidney disease (CKD) in North Indians.

## Materials and methods

**Study Population**

The study was carried out on 223 subjects comprising 123 unrelated ADPKD cases and 100 healthy, unrelated controls (without any disease, including renal, enrolled from among those visiting the hospital). The patients of ADPKD were enrolled from the Nephrology Department of Sir Ganga Ram Hospital and those referred to the Genetics clinic of the hospital. The study was approved by the institutional ethics committee of the hospital vide letter no Ref: EC/10/10/199(A) and informed consent was obtained from all participants. The diagnosis was established on the basis of Ravine’s criteria for ADPKD (16,17) and glomerular filtration rate (eGFR) was estimated by modification of diet in renal disease (MDRD) equation using the value of serum creatinine. Based on these eGFR values, the ADPKD patients were initially categorized into five stages of CKD (1-5) and later re-grouped into two sub-stages: early (CKD 1-3) and advanced (CKD 4 & 5) (18).

A comparison of genotype and allele frequencies was made broadly among four groups: group 1 (patients with ADPKD), Group 2 (ADPKD patients with advanced CKD stage), group 3 (ADPKD patients with early CKD-stage), and group 4 (controls). ADPKD patients in group 2 (n=51) had either started dialysis or underwent kidney transplant or were in need of renal replacement therapy.

**Genotyping of VEGF promoter polymorphisms**

**Fig. 1:**
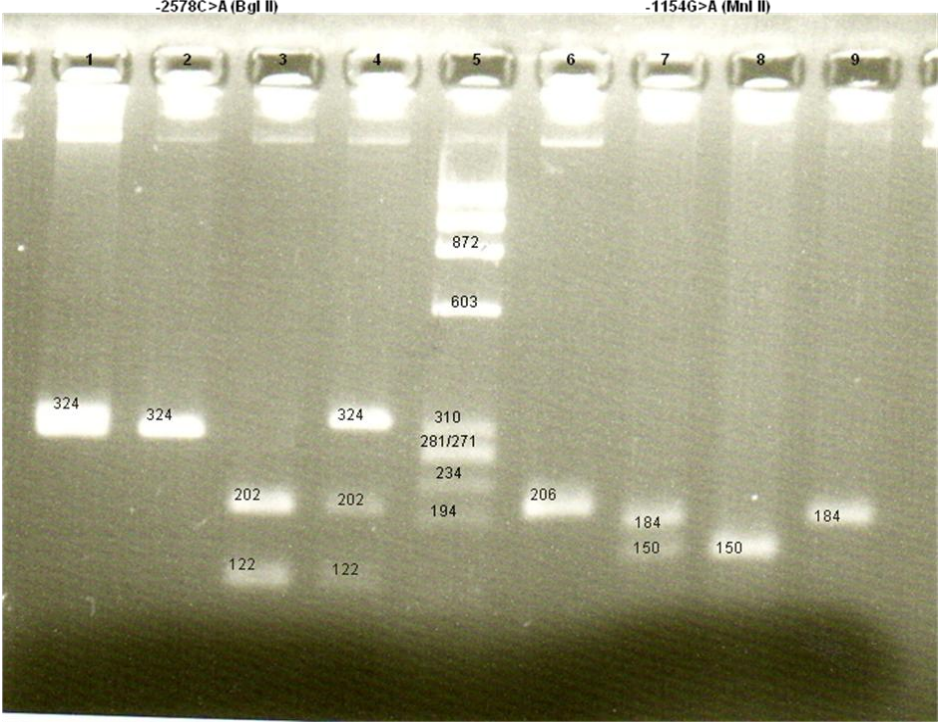
***VEGF*** **gene promoter -2578C>A and -1154G>A** **polymorphisms.**

DNA was extracted from peripheral blood(19) and amplified using previously published primers (13). PCR was carried out followed by restriction fragment length polymorphisms (RFLP) analysis to detect the alleles present in each subject. The PCR conditions were as follows: initial denaturation for 5 min at 95 C, followed by 35 cycles of denaturation at 94 C for 35 s, annealing at 60 C for 35 s, extension at 72 C for 40 s, and a final extension at 72 C for 10 min. RFLP analysis was used to detect the alleles present in each subject. The PCR products were digested overnight with appropriate restriction enzymes and the digested products were analyzed on a 3% agarose gel. For -2578C>A polymorphism study, Bgl II enzyme was used for digestion. Homozygous CC genotype showed a single band of 324 bp, homozygous AA genotype gave two bands of 202 and 122 bp and heterozygous CA genotype showed three bands of 324, 202 and 122 bp (Figure 1). For the -1154G>A polymorphism study, the PCR product was digested using Mnl I enzyme to give 3 bands of 150, 34 and 22 bp for homozygous GG genotype, two bands of 184, 22 bp for homozygous AA genotype, and 4 bands of 184, 150, 34 and 22 bp for heterozygous GA genotype (Figure 1).

Lane 1: Undigested PCR product for *VEGF* -2578C>A; lanes 2-4: RFLP for -2578C>A using Bgl II restriction enzyme; lane 5: size marker Ø174/Haem III; lane 6: Undigested PCR product for *VEGF* -1154G>A; lanes 7-9: RFLP for -1154G>A using Mnl II restriction enzyme. Lanes 2- 4 represent CC, AA, and CA genotypes, respectively. Lanes 7- 9 represent GA, GG, and AA genotypes, respectively.

**Statistical analysis**

Differences in the *VEGF* genotypes, allele frequencies and haplotypes among the four defined groups were analyzed using Fisher’s exact test or Chi-square (χ2) test. Hardy-Weinberg equilibrium (HWE) was evaluated for both cases and controls using Chi-square test and the goodness-of-fit test. The strength of association between genotypes, allele frequencies and haplotypes were measured using odds ratio (OR) (20). SNPStats was used to study different models of inheritance (21). Linkage disequilibrium and haplotypes were established using SHEsis software (22) and statistical analysis was done using microsoft excel and statistical package for social sciences program (SPSS) software Version 21. All P-values<0.05 were considered as statistically significant.

## Results

The mean age of the patients with ADPKD (group 1) was 41.54± 12.9 years with a male to female ratio equal to 1.32, and control group (group 4) had 38.88± 7.2 years with a male to female ratio equal to 0.69 (P=0.067). Patients belonging to advanced CKD stage (group 2) consisted of 69 cases with mean age of 45.24± 13.0 years and patients belonging to early CKD stage (group 3) consisted of 54 cases with mean age of 36.79± 11.29 years. The mean creatinine values for groups 2 and 3 were 607.31± 335.04 µmol/L and 87.52± 36.24 µmol/L, respectively. The healthy controls had a mean serum creatinine value of 78.68± 71.6 µmol/L.

**Distribution frequency of -2578C >A and -1154G> A polymorphisms**

The allele and genotype frequencies of the two promoter polymorphisms in different groups are shown in Table 1. Both polymorphisms were in HWE in control population, however, a slight deviation was observed in cases for -2578C>A (P= 0.049). A statistically significant difference in genotype frequencies was observed in ADPKD patients (group 1) and controls (group 4) for -2578C>A (P= 0.003), though allele frequencies did not differ significantly among the two groups (P= 0.491). In contrast, the allele and genotype frequencies for -2578C>A and -1154G>A did not differ significantly among cases with advanced (group 2) and early CKD stage (group 3).

Further evaluation was carried out under different models of inheritance (co-dominant, dominant, recessive and over- dominant) for both polymorphisms (23). Table 2 summarizes the results from these models and the association tests between polymorphisms studied and ADPKD. Under the recessive model (AA vs CC+CA), it was observed that the A allele of -2578C>A provided protection against ADPKD while C allele was a risk-associated allele (OR, 3.73; 95%CI, 1.45-9.62; P= 0.0042). This difference was also significant when healthy controls were compared with subgroups of ADPKD patients: ADPKD with early CKD stage (P= 0.032) and ADPKD with advanced CKD stage (P= 0.026), respectively. A significant difference (P= 0.0015) was also observed for CA heterozygotes under the overdominant model for the same polymorphism. It was observed that ADPKD-advanced CKD group had higher frequency of CA heterozygotes and the difference was significant when compared with controls (P<0.001).

**Table 1:** Distribution of *VEGF* genotypes and alleles frequencies in ADPKD patients and controls.

					**Group 1 vs Group 4**		**Group 2 vs Group 3**	
**Genotype/** **Allele**	**Group 1: ADPKD patients (n=123)**	**Group 2: ADPKD-Advanced CKD stage (n=69)**	**Group 3: ADPKD-Early CKD stage (n=54)**	**Group 4: Controls (n=100)**	**χ2** ** p-value**	**OR (95%CI)**	**χ2 p-value**	**OR (95%CI)**
rs699947 (-2578C>A)								
CC	0.309	0.275	0.352	0.37	0.003*		0.644	
CA	0.626	0.652	0.593	0.44				
AA	0.065	0.073	0.056	0.19				
C	0.622	0.601	0.648	0.59	0.491	0.87 (0.59-1.28)	0.454	1.22 (0.72-2.05)
A	0.378	0.399	0.352	0.41				
rs1570360 (-1154G>A)								
GG	0.431	0.42	0.444	0.57	0.093		0.862	
GA	0.48	0.478	0.481	0.34				
AA	0.089	0.101	0.074	0.09				
G	0.671	0.659	0.685	0.74	0.111	1.39 (0.92-2.11)	0.669	1.21 (0.65-1.92)
A	0.329	0.341	0.315	0.26				

* Statistically significant

For -1154G>A polymorphism, the frequency of G allele and GG genotype among the four groups was higher in controls (G, 74%; GG, 57%). The frequency distribution of homozygous GG genotype in advance-CKD stage and early-CKD stage cases was 42% and 44.4%, respectively. Under dominant model of inheritance (GA+AA vs GG), A allele was significantly associated with risk to ADPKD (OR, 0.55; 95%CI, 0.31-0.98; P= 0.041). This finding is also supported by the marginally significant association of GA heterozygotes with disease risk in the cases (OR, 0.53; 95%CI, 0.29-0.95; P= 0.031) and in advanced CKD group (OR, 0. 41; 95%CI, 0.19 - 0.87; P=0.018).

**Linkage disequilibrium and haplotype analysis**

Haplotypes were constructed for the two polymorphisms (-2578C>A and -1154G>A) using SHEsis software (22) to evaluate the combined effect of both polymorphisms. The two polymorphisms were in strong linkage disequilibrium in cases, but weakly in controls (D’ = 0.845, r^2^ = 0.577; D’ = 0.802, r^2^ = 0.326) (24). There were four common haplotypes [A;A], [A;G], [C;A] and [C;G], and their distribution was similar between the cases and the controls. Haplotype C;G was the most common haplotype with frequency of 59% and 56% in cases and controls, respectively. The global result for group 1 (ADPKD cases) and group 4 (controls) for the haplotype block (rs699947 and rs1570360) was: total cases= 246, total controls= 200, global χ^2 ^=10.876, while df= 3, Fisher’s P= 0.0125, and Pearson’s P=0.0125. Global haplotype association analysis showed [A;G] as a protective haplotype associated with ADPKD (OR, 0.398; 95%CI, 0.222-0.713; Fisher’s P=0.002; Pearson’s P=0.002). For group 2 (advanced-CKD stage), and group 3 (early-CKD stage) haplotypes were constructed and the global results were: total cases with advanced-CKD stage= 138, total cases with early-CKD stage= 108, global χ^2 ^= 6.711, while df= 3, Fisher’s P= 0.0818, and Pearson’s P = 0.0817. There was no significant difference in the haplotype distribution between groups 2 and 3 (Table 3).

**Table 2:** Analysis by genetic models in cases (ADPKD) and controls (adjusted by age and sex)

		**Group 1: ADPKD patients (n=123)**	**Group 2: Advanced CKD stage (n=69)**	**Group 3: Early CKD stage (n=54)**	**Group 4: Controls (n=100)**	**Group 1 vs Group 4**		**Group 2 vs Group 4**		**Group 3 vs Group 4**		**Group 2 vs Group 3**	
rs699947 (-2578C>A)	Genotype	Frequency (%)	frequency (%)	frequency (%)	frequency (%)	OR	p-value	OR	p-value	OR	p-value	OR	p-value
						(95%CI)		(95%CI)		(95%CI)		(95%CI)	
Codominant	C/C	38 (30.9)	19 (27.5)	19 (35.2)	37 (37.0)	1 (Ref)		1		1		1	
	C/A	77 (62.6)	45 (65.2)	32 (59.3)	44 (44.0)	0.49	0.0015*	0.23	<0.001*	0.73	0.072	0.60	0.52
						(0.25-0.93)		(0.09-0.59)		(0.33-1.57)		(0.25-1.45)	
	A/A	8 (6.5)	5 (7.3)	3 (5.6)	19 (19.0)	2.42		0.51		3.13		0.61	
						(0.87-6.74)		(0.17-1.58)		(0.76-12.88)		(0.11-3.26)	
Dominant	CC	38 (30.9)	19 (27.5)	19 (35.2)	37 (37.0)	1		1		1		1	
	CA+AA	85 (69.1)	50 (40.7)	35 (28.5)	63 (63.0)	0.66	0.18	0.36	0.015*	0.94	0.87	0.60	0.25
						(0.36-1.22)		(0.15-0.84)		(0.45-1.98)		(0.25-1.43)	
Recessive	CC+CA	115 (93.5)	64 (92.7)	51 (94.4)	81 (81.0)	1		1		1		1	
	AA	8 (6.5)	5 (7.3)	3 (5.6)	19 (19.0)	3.73	0.0042 *	3.63	0.026 *	3.76	0.032 *	0.86	0.85
						(1.45-9.62)		(1.08-12.16)		(0.98-14.38)		(0.18-4.12)	
Overdominant	CC+AA	46 (37.4)	24 (34.7)	22 (40.7)	56 (56.0)	1		1		1		1	
	CA	77 (62.6)	45 (65.2)	32 (59.3)	44 (44.0)	0.39	0.0015 *	0.20	<0.001*	0.56	0.12	0.66	0.32
						(0.21-0.70)		(0.09-0.48)		(0.27-1.17)		(0.29-1.50)	
rs1570360 (-1154G>A)													
Codominant	G/G	53 (43.1)	29 (42.0)	24 (44.5)	57 (57.0)	1 (Ref)		1		1		1	
	G/A	59 (48.0)	33 (47.8)	26 (48.1)	34 (34.0)	0.51	0.089	0.39	0.059	0.57	0.34	0.87	0.89
						(0.28-0.94)		(0.18-0.87)		(0.27-1.21)		(0.38-1.99)	
	A/A	11 (8.9)	7 (10.1)	4 (7.4)	9 (9.0)	0.80		0.80		0.76		0.71	
						(0.27-2.35)		(0.19-3.31)		(0.20-2.92)		(0.16-3.26)	
Dominant	GG	53 (43.1)	29 (42.0)	24 (44.5)	57 (57.0)	1		1		1		1	
	GA+AA	70 (56.9)	40 (58.0)	30 (55.5)	43 (43.0)	0.55	0.041*	0.44	0.03 *	0.60	0.16	0.85	0.68
						(0.31-0.98)		(0.21-0.94)		(0.29-1.23)		(0.38-1.88)	
R0ecessive	GG+GA	112 (91.1)	62 (89.9)	50 (92.6)	91 (91.0)	1		1		1		1	
	AA	11 (8.94)	7 (10.1)	4 (7.4)	9 (9.0)	1.09	0.87	1.23	0.77	0.97	0.97	0.77	0.72
						(0.39-3.80)		(0.32-4.77)		(0.27-3.56)		(0.18-3.29)	
Overdominant	GG+AA	64 (52.0)	36 (52.2)	28 (51.9)	66 (66.0)	1		1		1		1	
	GA	59 (47.9)	33 (47.8)	26 (48.1)	34 (34.0)	0.53	0.031*	0.41	0.018 *	0.59	0.16	0.92	0.83
						(0.29-0.95)		(0.19-0.87)		(0.29-1.22)		(0.42-2.03)	

OR: odds ratio; CI: confidence interval;

* statistically significant

**Table 3:** VEGF haplotype frequencies and the association with disease progression in different groups.

			**Group 1 vs Group 4**							**Group 2 vs Group 3**				
**Haplotype**	**Group 1: ADPKD patients (n=123)**	**Group 4: Controls (n=100)**	**χ2**	**Fisher's P**	**Pearson's P**	**OR**	**95% CI**	**Group 2: Advanced CKD stage (n=69)**	**Group 3: Early CKD stage (n=54)**	**χ2**	**Fisher's P**	**Pearson's P**	**OR**	**95% CI**
**AA**	0.298	0.23	2.599	0.107	0.107	1.421	0.926-2.180	0.29	0.305	0.064	0.8	0.8	0.931	0.54-1.61
**AG** ^#^	0.08	0.18	10.044	0.002	0.002	0.398	0.222-0.713	0.108	0.047	3.069	0.079	0.079	2.473	0.87-7.01
**CA**	0.032	0.03	0.007	0.935	0.935	1.046	0.356-3.076	0.05	0.01	3.167	0.075	0.075	5.35	0.68-41.90
**CG**	0.59	0.56	0.423	0.515	0.515	1.133	0.777-1.653	0.551	0.638	1.908	0.167	0.167	0.695	0.42-1.17

**Table 4:** Age (in years) at respective CKD stages and genotypes of *VEGF* polymorphisms

**rs699947 (-2578C>A)**	**CC+CA**	**n**	**AA**	**n**	**P value** *
Advanced CKD (n=69)	45.01±12.8	64	50.2±11.4	5	0.314
Early CKD (n=54)	36.76±11.6	51	37.33±2.10	3	0.473
**rs1570360 (-1154G>A)**	**GA+AA**	**n**	**GG**	**n**	**P value** *
Advanced CKD (n=69)	44.07±11.4	40	47.21±14.4	29	0.422
Early CKD (n=54)	37.66±11.2	30	35.71±11.6	24	0.448

* Mann-Whitney U test

Logistic regression analysis was used to assess the relation between CKD progression and other independent variables among the ADPKD patients sub-groups 2 (advanced CKD) and 3 (early CKD). It was observed that the male gender (OR, 3.67; 95%CI, 1.6-8.3; P= 0.002) and a positive family history (OR, 3.12; 95%CI, 0.135-0.762; P= 0.010) of the disease were associated with a 3-fold increased risk of CKD progression.

Table 4 describes the age at respective CKD stages and the genotype present in ADPKD patient sub-groups for the present study. A statistically significant difference in the age at respective CKD stage was not observed with regard to different genotypes.

## Discussion

The results observed in the present case-control study demonstrate that the *VEGF* polymorphism may accelerate the progression of disease in ADPKD when compared to healthy controls. The allelic and genotypic distribution of the two studied *VEGF* polymorphisms in the present representative control population, is in concordance with South Asians data (SAS) of 1000 genomes (http:// phase3browser. 1000 genomes. org/ index. html; last accessed on 7^th^ May, 2017). Allele frequencies did not differ significantly between the two groups, but the frequency of A allele (-2578C>A) and G allele (-1154G>A) was higher in controls than cases, suggesting this may have a protective role in ADPKD cases. The above observation was also supported by the analysis of haplotypes where a statistically significant likely protective effect was observed for the *VEGF* gene haplotype [A;G] (frequency in cases vs. controls: 8% vs. 18%, respectively; P=0.002) (Table 3). However, with respect to cases in early and advanced-CKD stages, the difference between the genotypes and the haplotypes were not significant. Our results are consistent with those reported by Prakash et al. (25), who studied ESRD cases (n=300) with primary kidney disease such as chronic glomerulonephritis (CGN), chronic interstitial nephritis (CIN) and hypertensive nephrosclerosis (HTN). They evaluated the association of *VEGF* polymorphisms (-1154G>A, -2578C>A, +936C/T and 2549ID) with risk to ESRD. They observed that homozygous AA genotype of -1154G>A had three-fold risk association with ESRD in CGN and 1.49-fold risk association with allele A of this polymorphism in HTN cases. For -2578C>A polymorphism, CC and CA genotypes were associated with disease risk in CGN cases as well as in HTN cases. In our study, under recessive model, it was observed that for -2578C>A, carriers of C allele (CC+CA genotypes) may have a 3.7-fold CKD risk compared to AA genotypes. To calculate the risk for CKD in cases with no *VEGF* -1154 GG genotypes, we considered the homozygous GG as reference. About 1.8-fold increase in risk to progression to CKD was observed in carriers of GA or AA genotypes (95% CI: 1.03-2.98, P= 0.041).

Earlier Reiterová et al. (15) also observed that ADPKD patients (n=283) with AA genotype (-2578C>A) had better prognosis (42.7±3.5 years), as compared to those with CC+CA genotypes (40.5±3.8 years); and the [C;G] haplotype was significantly associated with earlier onset of ESRD in these patients. In our cohort, although the difference was not statistically significant, but the carriers of wild type homozygous AA genotype of -2578C>A in both groups and the carriers of GG genotype of -1154G>A in advanced-CKD group showed a delayed progression to the respective CKD stages (Table 4). A statistically significant association of [C;G] haplotype with progression to disease was, however, not observed in our study. This deviation could be due to the low frequency of subjects with AA genotype (8 and 11 in cases, 19 and 9 in controls, respectively) for these two polymorphisms.

Studies investigating the role of *VEGF-*polymorphisms as a susceptibility risk factor for progression of disease have shown variable results in different diseases. The CA and CC genotypes of -2578C>A polymorphism has been reported to be associated with increased risk for prostate cancer in Mexican males (26), in contrast to increased risk associated with AA genotype and A allele in patients with renal cell carcinoma in Chinese patients (27). The-1154AA genotype in Turkish females has been documented with higher risk of recurrent pregnancy loss (13), and increased risk of epithelial ovarian cancer in Indian women (28). The two polymorphisms have been reported to have no association with risk of disease in lung cancer in Chinese (14) and Japanese end stage renal disease patients (29). These discrepancies could be due to the ethnicity difference or may result from different impacts of *VEGF* polymorphisms in disease pathophysiology. In conclusion, the present study stipulates that allele A of -2578C>A and allele G of -1154G>A may modify the progression of disease in ADPKD, suggesting a likely protective effect for disease up to 70% and 45% of subjects studied, respectively. The [A;G] haplotype may provide a 2.5-fold protective effect for disease progression. In addition, male gender and positive family history might also increase the risk of CKD progression among patients of ADPKD. The limitations of the present study are the small sample size and that only two polymorphisms of *VEGF* gene were studied. Therefore, additional studies with larger sample sizes are necessary to confirm these preliminary results. Inclusion of other *VEGF* associated polymorphisms and investigating their combined effect would help to better understand their role as predisposing factors for progression of disease. Moreover, in the era of precision medicine, the identification of such variants, with respect to ethnicity and diseases, could provide information about targets for intervention to delay progression of disease.
